# Study on Gastrointestinal Nematodes of Equines in and around Bekoji, South Eastern Ethiopia

**DOI:** 10.1155/2022/8210160

**Published:** 2022-05-30

**Authors:** Mesfin Mathewos, Dereje Teshome, Haben Fesseha

**Affiliations:** ^1^School of Veterinary Medicine, Wolaita Sodo University, Wolaita Sodo, Ethiopia; ^2^Lemuna Bilbilo Livestock and Fishery Developmental Sector, Arsi Zone, Ethiopia

## Abstract

Parasitic infections can cause a variety of respiratory, intestinal, and other problems in horses, as well as contribute to some performance issues. A cross-sectional study was undertaken in and around Bekoji, South Eastern Ethiopia, from November 2020 to June 2021 to identify species and evaluate the prevalence of gastrointestinal parasites in donkeys and horses, using direct fecal smear, floatation methods, and larval cultures. In this study, the overall prevalence of gastrointestinal nematode parasites was 94.5% (363 out of 384), with donkeys accounting for 95.8% and horses accounting for 90.5%. The coprological study indicated that an overall prevalence of gastrointestinal nematode parasites was 87%, 8.3%, 2.5%, 1.4%, and 0.8% for mixed parasite infection, nonmigratory strongylids, migratory strongylids, *Parascaris equorum*, and *Oxyuris equi*, respectively. Among mixed infections, nonmigratory strongylids+migratory strongylids (51.5%) occurred most frequently. The odds of male horses being infected by GIT nematodes were 1.59 times higher than male donkeys. Horses which have poor body condition were 2.94 times more infected than donkeys. The odds of old-aged donkeys were 3.11 times more infected than horses. A statistically significant difference (*p* < 0.05) was observed in the prevalence of gastrointestinal nematodes with species and body conditions of the animals. However, no statistically significant difference (*p* > 0.05) was seen in the prevalence of gastrointestinal nematode parasites between the sex and age of the Equidae. The mean fecal egg count of nematodes revealed that horses (1364.4 ± 483.5) had a more severe infection than donkeys with a statistically significant difference (*p* < 0.05). The current study determined there was a high prevalence of gastrointestinal nematode parasites in Equidae. Regular deworming, improved housing and nutrition management systems, increased animal owner awareness, and prevention techniques should all be undertaken to minimize the disease's economic burden in the area.

## 1. Introduction

Ethiopia is a developing country in Africa that is mostly an agricultural country, with over 85% of its population working in the agricultural sector [1, 2]. With an estimated 190.93 million tropical livestock units, including 7.04 million donkeys, 2.03 million horses, and 0.4 million mules, the country has the greatest livestock population among African countries [3].

Equidaes are widely used as working animals in various regions of the world, where they are used for packing, riding, hauling, and plowing [4, 5]. Equidae power is essential for both rural and urban transportation systems that are inexpensive and give the best options in locations where the road network is underdeveloped, the terrain is steep and mountainous, and in cities where tight streets make it difficult to convey goods. Donkeys, it is proposed, can play a significant role in the frameworks of food security and social fairness in nations with high levels of food insecurity [6]. Many people utilize Equidaes to bring food and other goods to villages in locations where there are no roads. Horses and donkeys are subjected to long working hours and tough conditions. When these animals are not working for long periods, they are left to wander and graze on waste. These have the potential to hurt their well-being and health [7]. Horses and mules are more powerful and faster-working animals, but they are more expensive to buy and keep than donkeys [8].

Despite its vast population, Equidae remains marginal because of a high frequency of hunger, management constraints, and diseases such as parasites. Reduced power output, diarrhea, colic, emaciation, impaired growth, poor reproductive performances, short lifespan, susceptibility to other infectious diseases, and high morbidity and mortality rates are just a few of the indicators of parasitism, which is a major impediment to the country's livestock farming system's development [9–11].

Helminthes in working Equidaes are extremely common; infection intensities are extremely high, and they are the leading cause of Equidae deaths in the country [12, 13]. Migratory strongylids, nonmigratory strongylids, *Parascaris equorum*, and *Oxyuris equi* are the most prevalent gastrointestinal nematode parasites of Equidae [14]. Gastrointestinal nematodes are a major barrier to Equidae growth and productivity in Ethiopia, as well as many other African countries. Apart from a few studies in other parts of Ethiopia, there is a scarcity of detailed and up-to-date information on gastrointestinal nematodes in donkeys and horses in and around Bekoji. The goal of this study was to determine the prevalence of gastrointestinal nematode infections in horses and donkeys in and around Bekoji, Ethiopia, as well as the risk factors associated with it.

## 2. Material and Methods

### 2.1. Study Area

The study was conducted from November 2020 to June 2021 in and around Bekoji, South Eastern Ethiopia. It is located about 222 km southeast of Addis Ababa and 56 km south of Asela. Located in the Arsi Zone of the Oromiya regional state of Ethiopia, it has a latitude and longitude of 7∗35′N39∗10∗E with an elevation of 2810 m. It is the administrative center of Limuna Bilbilo woreda. The area has three distinct seasons, namely, main rainy (June to September), short rainy (March to May), and dry (October to February) seasons. The production system of the study area was predominantly mixed crop–livestock activities.

### 2.2. Study Animal

The donkeys and horses including all age and sex groups managed under a smallholder mixed crop–livestock farming system were included in the study. The age of the study animals was determined based on the owners' information and dentition. The Equidaes were grouped into three age categories as young (<2 years), adult (2-10 years), and old (>10 years) [15] whereas the body condition score was described by [16].

### 2.3. Study Design

A cross-sectional study was conducted from November 2020 to June 2021 to determine the prevalence of gastrointestinal nematode infections in horses and donkeys in and around Bekoji, Ethiopia, as well as the risk factors associated with them.

### 2.4. Sample Size

A simple random sampling technique was used to select study animals. The sample size was determined using the formula given by Thrusfield et al. [17] with 50% expected prevalence, a 5% desired absolute precision, and a 95% confidence interval. Therefore, 384 (289 donkeys and 95 horses) Equidaes would be obtained for the study using the formula.

(1) $$\begin{array}{c} \frac{N=Z^{2}Х\text{ Pexp }\left(1-\text{Pexp}\right)}{d^{2}}, \end{array}$$

where *N* is the required sample size, *d* is the desired absolute precision = 0.05, *Z*^2^ is the statistic for level of confidence = 1.96, and Pexp is the expected prevalence.

### 2.5. Parasitological Analysis

Fecal samples were collected directly from the rectal ampulla of each donkey and horse using disposable arm-length gloves and stored in the universal bottle in the veterinary parasitology laboratory at Bekoji Agricultural Technical Vocational Education and Training College until evaluation. The simple flotation test was used to identify the nematode eggs based on their size, shape, color, content (embryo/larvae), and absence of operculum, as previously described by Hendrix and Robinson [18], and the McMaster method [19] was used to determine egg per gram of feces (EPG) according to Nielsen et al. [20] and Kaplan and Nielsen [21]. Accordingly, the Equidaes are categorized into mild if the egg count level is within the range of 0–799 EPG, moderate if the egg count level is 800–1200 EPG, and severe if the egg count level is greater than 1200 EPG [22, 23]. About 10 g of feces was placed on a gauze stretched within a plastic cup containing about 20 mL of water for the fecal cultures, which were then covered with the bottom of a perforated plastic cup to provide appropriate oxygenation. To allow complete larval development, fecal cultures were maintained at 27°C for 7-10 days. To maintain proper humidity levels during the incubation period, cultures were sprayed with distilled water. L3 were obtained using the Baermann procedure [24] after the incubation period. Harvested L3 from each culture were evaluated microscopically and identified using two previously published morphological identification keys based on total body length and width, intestinal and oesophageal length, and the quantity, arrangement, and form of intestinal cells (IC) [20, 25, 26].

### 2.6. Data Analysis

The summary of laboratory and field data was presented in Microsoft Excel spreadsheets. Descriptive statistics and chi-square were computed to determine the prevalence of nematode infection among Equidaes while the independent *t*-test statistic was calculated to compare the mean of EPG between species, sex, body condition, and age of Equidae using the STATA version 13 statistical software. Statistical significance was set at *p* ≤ 0.05, and the results were presented in tables and graphs.

### 2.7. Ethical Statement

This study received ethical approval from the Wolaita Sodo University of Research Ethics and Review Committee. Before taking samples, Equidae owners were asked for verbal authorization to obtain samples from their horses and donkeys under stringent hygienic conditions. The goal of the study was followed according to best practice recommendations for animal care, and the Wolaita Sodo University of Research Ethics and Review Committee authorized the oral informed consent process.

## 3. Results

### 3.1. Prevalence and Associated Risk Factors of GIT Nematodes

The overall prevalence of GIT nematode in donkeys and horses was 94.5% (*n* = 363/384). Up on chi-square analysis, putative risk factors such as body condition and species of Equidae revealed a statistically significant association (*p* < 0.05) with the occurrence of GIT nematode infection. Moreover, no statistically significant variation was seen in the prevalence of GIT nematode infection among age and sex groups (Table 1).

This study revealed that the odds of male, old, and medium body conditioned donkeys infected with GIT nematodes were 0.567, 3.11, and 1.38 times higher than female, young, and poor body conditioned donkeys, respectively. On the other hand, the odds of male, young, and poor body conditioned horses infected with GIT nematodes were 1.59, 0.45, and 2.94 times higher than female, old, and medium body conditioned horses, respectively, (Table 2). Although a statistically significant difference (*p* < 0.05) was observed with the prevalence of GIT nematodes between sex and age of donkeys but not observed with body conditions. However, in the case of horses, a statistically significant variation (*p* < 0.05) was observed in the prevalence of GIT nematodes between age groups.

In this study, Equidae infected by mixed infection (87%) of GIT nematode parasites indicated the highest prevalence followed by nonmigratory strongylids (8.3%), migratory strongylids (2.5%), *P. equorum* (1.4%), and *O. equi* (0.8%). Species, body condition, and age of Equidae have shown a statistically substantial difference (*p* < 0.05) with the occurrence of mixed parasite infection. Body condition and age have also revealed a statistically significant difference (*p* < 0.05) with the occurrence of nonmigratory strongylids but in the case of *O. equi*, only age has shown a statistically significant effect (*p* < 0.05) (Table 3).

In this study, horses and donkeys were highly infected by mixed infection (79.1% and 89.5%) of GIT nematode parasites followed by nonmigratory strongylids (10.4% and 7.5%), respectively. The sex and age of horses have shown a statistically substantial difference (*p* < 0.05) with the prevalence of GIT nematode parasites. However, in donkeys, statistically significant difference (*p* < 0.05) was observed between body condition and age of donkeys with the prevalence of GIT nematode parasites (Table 4).

## 4. Frequency and Percentage of Mixed Parasite Infection

**Figure 1:**
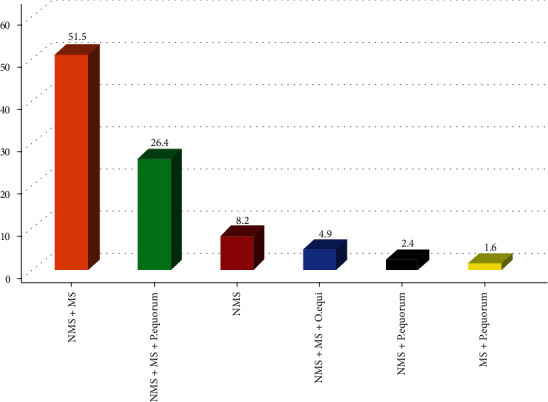
Mixed nematode parasites.

Among mixed infestation, nonmigratory strongylids+migratory strongylids (51.5%) occurred the most frequently followed by nonmigratory strongylids+migratory strongylids+Parascaris equorum (26.4%) (Figure 1).

### 4.1. Correlation Analysis of GIT Nematode Parasites

Among the associated risk factors, the body condition of the Equidaes had strong a positive correlation (*r* = 0.4057) with the prevalence of GIT nematode infestation. However, the risk factors such as species (*r* = −0.1562) and sex (*r* = −0.0170) had a weak negative correlation with the prevalence of GIT nematode parasites (Table 5).

### 4.2. Larval Recovery and Fecal Egg Count of GIT Nematode Parasites

No nematodes other than migratory Strongylus (Strongylus) and nonmigratory Strongylus (Cyathostomin) infective larvae (L3) were recovered from the samples in the studies. The mean fecal egg count of nematodes revealed that horses (1364.4 ± 483.5) had a more severe infection than donkeys with a statistically significant difference (*p* < 0.05). Among risk factors, females (725.5 ± 259.7), good body condition (139.1 ± 119.5), and old (129.2 ± 87.07) Equidaes have had a light EPG. Equidaes which have poor body condition (853.4 ± 306.2) had a moderate EPG (Table 6).

## 5. Discussion

Gastrointestinal (GI) parasite infection has a direct impact on the health and productivity of working Equidae, resulting in a decline in output and, as a result, a reduction in the owner's and community's income [27, 28]. The overall prevalence of gastrointestinal parasites in the current study was 94.5% (95.8% in donkeys and 90.5% in horses), which was similar to previous reports from Gondar town by [29] who reported a prevalence of 95.8%, but higher than reports of [30–32] who indicated an overall prevalence of 76.04%, 72.7%, and 70.4%, respectively.

The high prevalence of GIT nematodes in donkeys was consistent with the findings of [1, 10, 28, 33–38] who stated a prevalence of 100%, 100%,100%, 92.8%, 100%,95.4%, 96.9%, 97.1%, and 97.13%, respectively, from different parts of the country, but higher than the reports of [30, 39] who described a prevalence of 86.5% and 37.48%, respectively. The higher parasitism seen in donkeys could be attributable to the fact the feeding habits (pasture grazing), lack of deworming, inadequate management, and not providing donkeys with animal welfare (lack of due attention) might provoke to serious changes in the gastrointestinal microbiota that result in dysbiosis [38].

The occurrence of GIT nematodes in horses was similar to [28] who reported 89.7% but lower than those [35] who observed a prevalence of 100%. However, [10, 38, 40] found a lower prevalence of 81%, 80.95%, and 65.51%, respectively. Because all of the research horses in the study area were cart horses that were fed grain byproducts and were not exposed to pasture grazing, they had a higher prevalence of GIT nematode infections than horses. [41]. The discrepancies of the present finding as compared to others could be due to the differences in the management systems, sample sizes, deworming strategy, the availability of veterinary clinic, and the nutritional state of the animals in each study area [10, 30].

Putative risk factors including species and body conditions of Equidaes have shown a statistically significant difference (*p* < 0.05) with the occurrence of GIT nematodes and was consistent with previous results [1, 10, 14, 31, 37]. As previously described by [1, 10, 37], animals with poor body conditions have a higher prevalence of helminth parasites than those which are well-conditioned and have found to be in line with the present study. The high prevalence of GIT nematodes in animals with poor body conditions could be related to expanded agricultural acreage, which confines animals to tiny community grazing areas, allowing for constant exposure [37]. However, there was no statistically significant variation in the prevalence of gastrointestinal parasites across age and sex of animals, which was consistent with other Ethiopian investigations [10, 42].

In the present study, a mixed presence of parasites comprising double or triple types was encountered in 87% of Equidaes which were higher than the finding of [43] in the Arsi-Bale highlands of the Oromia Region, [44] in Turkey, [45] in Ambo town, [28] in and around Guder town, and [46] in Pakistan, who reported a prevalence of 59.1%, 50%, 25%, 13.8%, and 5%, respectively. This could be attributed to management variances, deworming activities, and seasonality. In this investigation, nonmigratory strongylids+migratory strongylids (51.5%) were shown to be the most predominant mixed parasite infections followed by nonmigratory strongylids+migratory strongylids+*Parascaris equorum* (26.4%), which was consistent with a previous finding of [32]. This could be due to their global spread and the fact that they are the most pathogenic nematode parasites of Equidaes that were found anywhere following the availability of grasslands [47]. The challenge of mixed infections exacerbates the compromised health condition of the animal which could result in debilitation and death of the animal [48].

This study revealed that an overall prevalence of migratory strongylids was 2.5% (4.6% in horses and 1.8% in donkeys) which accords with [35]. On the other hand, it is due to its large numbers of genera and species so the percentages of migratory strongylids usually constituted 75-100% of whole nematode infections [49], which contradicted the current data. The prevalence of migratory strongylids in horses was lower than the reports of [2, 3, 10, 31, 32, 36, 38, 40, 44, 50–53] who reported a prevalence of 58.50%, 66.7%, 91%, 99%, 100%, 66.6%, 76%, 58.5%, 68%, 63.7%, 45.1%, 48.2%, 48.2%, and 4.92%, respectively, in horses. The decreased prevalence of migratory strongylids in the horses may be related to the fact that all of the study horses were cart horses, which are less exposed and, in some circumstances, limited from pasture. The prevalence of migratory strongylids in donkeys wase lower than the previous findings of [1–3, 7, 10, 31–34, 36, 38, 42, 44, 50, 52–57], who indicated a prevalence of 5.82, 44.55%, 59.1%, 60.6%, 66.07, 70.8%, 76%, 79.7%, 80.2%, 81%, 82.75%, 87.81%, 87.8%, 88.21%, 95.5, 99%, 100%, 100%, 98.2%, 100%, and 100%, respectively. Strongyle infections are more common, which is consistent with their biology and epidemiology, as these parasites take longer to complete their life cycle and their burden has fluctuated over time due to anthelminthic stresses [14]. Furthermore, the overall prevalence of nonmigratory strongylids was 8.3% (10.4% in horses and 7.5% in donkeys). This finding agreed with Reinemeyer et al. [58] who have been reported that the overall prevalence of nonmigratory strongylids is high in horses, which is believed that 100% of horses are infected with these parasites. The low prevalence of nonmigratory strongylids in donkeys of the current study was in line with previous reports of [59, 60] who stated that only a small proportion of animals have patent nonmigratory strongylid infections.

The overall prevalence of *Parascaris equorum* was 1.4% (3.4% in horses and 0.7% in donkeys), which was lower than the previous works of [6, 15], who reported an overall prevalence of 15.7% and 17.1% from the highlands of Wollo provinces and western highlands of Oromia, respectively. The current study's lower prevalence of *Parascaris equorum* in horses contradicts reports by [13, 33, 36] who reported 15.7%, 7.3%, and 16.2%, respectively, from various parts of Ethiopia, and by [61] who reported 21.6% in Lesotho. In the current study, the prevalence of *Parascaris equorum* in donkeys was again lower than reports of [1, 10, 13, 33, 36, 50, 62] who indicated a prevalence of 15.7%, 17.3%, 42.29%, 43.5%, 50%, 51%, and 51%, respectively. The prevalence of *Parascaris equorum* reported in different studies in underdeveloped nations is somewhat contradictory, and this could be related to weakened immune responses due to concomitant infection, but it warrants more exploration [61].

*Oxyuris equi* (0.8%) was one of the least prevalent parasites isolated in horses (2.3%) and donkeys (0.4%) in the current investigation. This overall prevalence was lower than the work of [15] who reported 32.4%. The significant prevalence of *Oxyuris equi* in donkeys contradicted the findings of [1, 10, 50] who found a prevalence of 4.3%, 3%, and 2%, respectively. The prevalence of *Oxyuris equi* in horses agreed with the finding of [10] who reported a prevalence of 0.95% in the Western highlands of Oromia but lower than [36, 61] who observed a prevalence of 2.1% around Gondar and 6.2% in Lesotho, respectively. The lowest occurrence could be attributed to the effect of the season when the sample was taken and the technique was utilized, as *Oxyuris equi* eggs were identified in the feces less frequently.

Fecal egg count is a vital index in the epizootiology of nematodes; it indicates the extent and intensity of parasitism and the importance of pasture contamination in the transmission of parasites [22]. The pathogenicity of Strongyles and other nematodes is related to their fecundity and host resistance [63]. Thus, the presence of severe overall mean EPG (1364.4 ± 483.5) in horses of the current as compared to donkeys (641.4 ± 250.5) was in line with the previous works of [57]. On the other hand, [64, 65] have been found to cope with high FEC (>1026.9) and (>3000 EPG) without a significant impact on health or productivity in donkeys, respectively. The high proportion of light infection observed among the infected donkeys indicates low worm burdens or the presence of active protection and suggests that donkeys may serve as reservoir hosts for other susceptible equids in the study area [57]. Age, sex, and body condition differences in the prevalence and intensity of gastrointestinal parasites in the current study were found to have concurred with the previous reports of [64, 66] in Nigeria.

## 6. Conclusion

Gastrointestinal parasites are one of the most common factors that constrain the health and working performance of donkeys and horses. The present study suggested a high prevalence of gastrointestinal nematodes in donkeys as compared with horses in the study areas. Mixed parasite infestation, nonmigratory strongylids, migratory strongylids, *P. equorum,* and *O. equi* were the major species of parasite that were identified in the study period. Among the putative risk factors, species and body conditions of Equidaes were shown a statistically significant difference with gastrointestinal nematode infection. Thus, a regular and strategic deworming program with efficacious anthelminthic and improving husbandry practice systems should be warranted to minimize the associated impacts on the economy and productivity of donkeys and horses in the study area.

## Figures and Tables

**Table 1:** Prevalence and associated risk factor of GIT nematodes of Equidae.

Risk factor		No. of examined	No. of positive (%)	*χ* ^2^	*p* value
Species	Donkey Horse	289 95	277 (95.8%) 86 (90.5%)	3.92	0.048
Sex	Male Female	188 196	175 (93.1%) 188 (95.9%)	1.49	0.222
BSC	Good Medium Poor	56 251 77	43 (76.7%) 243 (96.8%) 77 (100%)	41.1	0.001
Age	Young Adult Old	54 247 83	54 (100%) 229 (92.7%) 80 (96.3%)	5.25	0.072
Overall prevalence		384	363 (94.5%)		

**Table 2:** Logistic regression analysis reporting odds ratio in donkeys and horses.

Species	Risk factor		No. of positive	Prevalence	OR	(95% Conf. Interval)	*χ* ^2^	*p* value
Donkeys	Sex	Male Female	126 151	45.5% 54.5%	0.567 Ref	0.355-0.907 Ref	4.79	0.029
	BSC	Good Medium Poor	35 185 57	12.6% 66.8% 20.6%	Ref 1.38 1.25	Ref 0.73-2.59 0.55-2.82	3.03	0.220
	Age	Young Adult Old	162 71 44	58.4% 25.6% 15.8%	2.46 Ref 3.11	1.10-5.51 Ref 1.58-6.12	15.03	0.001
Horses	Sex	Male Female	49 37	56.9% 43.1%	1.59 Ref	0.96-2.62 Ref	2.85	0.091
	BSC	Good Medium Poor	8 58 20	9.3% 67.4% 20.2%	Ref 2.26 2.94	Ref 0.99-5.13 1.11-7.76	2.75	0.252
	Age	Young Adult Old	10 67 9	11.6% 77.9% 10.4%	0.45 Ref 0.28	0.19-1.02 Ref 0.13-0.60	10.01	0.007

**Table 3:** Species wise prevalence and their associated risk factors for the occurrence of GIT nematodes in Equidae.

Risk factors		Species of GIT nematode parasites identified									
		Migratory strongylids		Mixed infection		*O. equi*		*P. equorum*		Nonmigratory strongylids	
		Freq	*χ* ^2^ (*p*-*v*)	Freq (%)	*χ* ^2^ (*p*-*v*)	Freq (%)	*χ* ^2^ (*p*-*v*)	Freq (%)	*χ* ^2^ (*p*-*v*)	Freq (%)	*χ* ^2^ (*p*-*v*)
Species	Donkey	5 (55.6)	1.92 (0.16)	248 (78.5)	9.94 (0.002)	1 (33.3)	2.85 (0.09)	2 (40)	3.38 (0.066)	21 (70)	0.48 (0.487)
	Horse	4 (44.4)		68 (21.5)		2 (66.7)		3 (60)		9 (30)	
Sex	Female	2 (22.2)	3.06 (0.08)	165 (52.2)	0.98 (0.321)	2 (66.7)	0.29 (0.587)	4 (80)	1.70 (0.192)	15 (50)	0.01 (0.905)
	Male	7 (77.8)		151 (47.8)		1 (33.3)		1 (20)		15 (50)	
Body condition	Good	1 (11.1)	2.70 (0.25)	31 (9.8)	44.5 (0.001)	1 (33.3)	1.33 (0.513)	1 (20)	1.28 (0.526)	9 (30)	11.9 (0.003)
	Medium	8 (88.9)		208 (65.8)		2 (66.7)		4 (80)		21 (70)	
	Poor	0 (0)		77 (24.4)		0 (0)		0 (0)		0 (0)	
Age	Adult	8 (88.9)	2.91 (0.23)	189 (59.8)	16.6 (0.001)	0 (0)	10.9 (0.004)	4 (80)	0.91 (0.63)	28 (93.4)	11.9 (0.003)
	Old	0 (0)		75 (23.7)		3 (100)		1 (20)		1 (3.3)	
	Young	1 (11.1)		52 (16.5)		0 (0)		0 (0)		1 (3.3)	
Over all prevalence		9 (2.5)		316 (87)		3 (0.8)		5 (1.4)		30 (8.3)	

**Table 4:** Species wise prevalence and their associated risk factors for the occurrence of GIT nematodes.

Species of animal	Risk factors		Species of GIT nematode parasites identified in horses					*χ* ^2^ (*p* value)
			Migratory strongylids	Mixed infection	*O. equi*	*P. equorum*	Nonmigratory strongylids	
			Freq (%)	Freq (%)	Freq (%)	Freq (%)	Freq (%)	
Horse	Sex	Female	0 (0)	31 (45.6)	1 (50)	3 (100)	2 (22.2)	11.4 (0.043)
		Male	4 (100)	37 (54.4)	1 (50)	0 (0)	7 (77.7)	
	Body condition	Good	0 (0)	6 (8.8)	1 (50)	0 (0)	1 (11.1)	13.8 (0.182)
		Medium	4 (100)	42 (61.7)	1 (50)	3 (100)	8 (88.8)	
		Poor	0 (0)	20 (29.4)	0 (0)	0 (0)	0 (0)	
	Age	Adult	3 (75)	52 (76.5)	0 (0)	3 (100)	9 (100)	23.05 (0.011)
		Old	0 (0)	7 (10.3)	2 (100)	0 (0)	0 (0)	
		Young	1 (25)	9 (13.2)	0 (0)	0 (0)	0 (0)	
	Over all prevalence		4 (4.6)	68 (79.1)	2 (2.3)	3 (3.4)	9 (10.4)	
Donkeys	Sex	Female	2 (40)	134 (54)	1 (100)	1 (50)	13 (61.9)	6.54 (0.257)
		Male	3 (60)	114 (45.9)	0 (0)	1 (50)	8 (38.1)	
	Body condition	Good	1 (20)	25 (10)	0 (0)	1 (50)	8 (38.1)	22.9 (0.011)
		Medium	4 (80)	166 (66.9)	1 (100)	1 (50)	13 (61.9)	
		Poor	0 (0)	57 (22.9)	0 (0)	0 (0)	0 (0)	
	Age	Adult	5 (100)	137 (55.2)	0 (0)	1 (50)	19 (90.5)	33.5 (0.001)
		Old	0 (0)	68 (27.4)	1 (100)	1 (50)	1 (4.7)	
		Young	0 (0)	43 (17.3)	0 (0)	0 (0)	1 (4.7)	
	Over all prevalence		5 (1.8)	248 (89.5)	1 (0.4)	2 (0.7)	21 (7.5)	

**Table 5:** Correlation analysis.

Variables	Parasite	Species	Sex	BCS	Age
Parasite	1.0000				
Spp	-0.1562	1.0000			
Sex	-0.0170	0.1387	1.0000		
BCS	0.4057	-0.0123	0.0953	1.0000	
Age	0.2518	-0.1511	0.0750	0.3509	1.0000

**Table 6:** Fecal egg count of GIT nematode in Equidaes.

Variables		Number of infected (%)	EPG (mean ± SD)	*p* value
Spps	Donkey	277 (95.8%)	641.4 ± 250.5	0.02
	Horse	86 (90.5%)	1364.4 ± 483.5	
Sex	Female	175 (93.1%)	725.5 ± 259.7	0.27
	Male	188 (95.9%)	1018.1 ± 358.6	
BCS	Good	43 (76.7%)	139.1 ± 119.5	0.98
	Medium	243 (96.8%)	544.8.3 ± 189.05	
	Poor	77 (100%)	853.4 ± 306.2	
Age	Young	54 (100%)	191.8 ± 155.1	0.16
	Adult	229 (92.7%)	623.2 ± 246.1	
	Old	80 (96.3%)	129.2 ± 87.07	

## Data Availability

The data will be provided upon the request of the corresponding author.

## Abbreviations

GIT: Gastrointestinal tract
NMS: Nonmigratory strongylids
MS: Migratory strongylids
STATA: Statistics and data.
